# Supplementary material for: Spontaneous Coronary Artery Dissection as a Cause of Acute Myocardial Infarction in COVID-19 Patients: A Case Report and Review of the Literature
Source: Healthcare (Basel). 2024 Jan 16;12(2):214. doi: 10.3390/healthcare12020214 (PMC10815188; doi:10.3390/healthcare12020214)
Supplement: Supplementary file 1 [file healthcare-12-00214-s001.zip › healthcare-2768375-supplementary.pdf]

CARE case report guidelines

CARE Checklist of information to include when writing a case report

(cc) BY-NC-ND

Topic	Item	Checklist item description	Reported on Line
Title	1	The diagnosis or intervention of primary focus followed by the words "case report"	p.1, line 3
Key Words	2	2 to 5 key words that identify diagnoses or interventions in this case report, including "case report"	p.1, line 27-28
Abstract (no references)	3a	Introduction: What is unique about this case and what does it add to the scientific literature?	p.1, line 18-26
	3b	Main symptoms and/or important clinical findings	p.1, line 15-16
	3c	The main diagnoses, therapeutic interventions, and outcomes	p.1, line 13-26
	3d	Conclusion—What is the main "take-away" lesson(s) from this case?	p.1, line 19-26
Introduction	4	One or two paragraphs summarizing why this case is unique (**may include references**)	p.1-2, line 31-46
Patient Information	5a	De-identified patient specific information.	p.2, line 48-59
	5b	Primary concerns and symptoms of the patient.	p.2, line 48-59
	5c	Medical, family, and psycho-social history including relevant genetic information	p.2, line 48-59
	5d	Relevant past interventions with outcomes	
Clinical Findings	6	Describe significant physical examination (PE) and important clinical findings.	p.2, line 55-59
Timeline	7	Historical and current information from this episode of care organized as a timeline	✓
Diagnostic Assessment	8a	Diagnostic testing (such as PE, laboratory testing, imaging, surveys).	p.2, line 64-70
	8b	Diagnostic challenges (such as access to testing, financial, or cultural)	p.2, line 68
	8c	Diagnosis (including other diagnoses considered)	p.2, line 67
	8d	Prognosis (such as staging in oncology) where applicable	
Therapeutic Intervention	9a	Types of therapeutic intervention (such as pharmacologic, surgical, preventive, self-care)	p.3, line 78-90
	9b	Administration of therapeutic intervention (such as dosage, strength, duration)	p.3, line 88-90
	9c	Changes in therapeutic intervention (with rationale)	p.8, line 236-239
Follow-up and Outcomes	10a	Clinician and patient-assessed outcomes (if available)	p.3, line 91-100
	10b	Important follow-up diagnostic and other test results	p.3, line 94-100
	10c	Intervention adherence and tolerability (How was this assessed?)	p.3, line 91-100
	10d	Adverse and unanticipated events	
Discussion	11a	A scientific discussion of the strengths AND limitations associated with this case report	p.8
	11b	Discussion of the relevant medical literature **with references**.	p.5-7
	11c	The scientific rationale for any conclusions (including assessment of possible causes)	p.3-4, line 132-158
	11d	The primary "take-away" lessons of this case report (without references) in a one paragraph conclusion	p.7-8, line 254-263
Patient Perspective	12	The patient should share their perspective in one to two paragraphs on the treatment(s) they received	
Informed Consent	13	Did the patient give informed consent? Please provide if requested	Yes ☑ No ☐
